# Exploratory study into awareness of heart disease and health care seeking behavior among Emirati women (UAE) - Cross sectional descriptive study

**DOI:** 10.1186/s12905-017-0445-4

**Published:** 2017-09-26

**Authors:** Sarah Khan, Syed Adnan Ali

**Affiliations:** 1 College of Natural and Health Sciences, Zayed University, Dubai, United Arab Emirates; 2 Government Degree Science and Commerce College, Landhi Korangi 6, Karachi, Pakistan

**Keywords:** Awareness, Atypical symptoms, Gender differences, Health seeking behavior, Heart disease, UAE, Women

## Abstract

**Background:**

Cardiovascular disease was the leading cause of death among women in the United Arab Emirates (UAE) in 2010. Heart attacks usually happen in older women thus symptoms of heart disease may be masked by symptoms of chronic diseases, which could explain the delay in seeking health care and higher mortality following an ischaemic episode among women. This study seeks to a) highlight the awareness of heart diseases among Emirati women and b) to understand Emirati women’s health care seeking behaviour in UAE.

**Method:**

A cross sectional, descriptive study was conducted using a survey instrument adapted from the American Heart Association National survey. A convenience sample of 676 Emirati women between the ages of 18–55 years completed the questionnaire.

**Results:**

The study showed low levels of awareness of heart disease and associated risk factors in Emirati women; only 19.4% participants were found to be aware of heart diseases. Awareness levels were highest in Dubai (OR 2.18, *p* < 0.05) among all the other emirates and in the 18–45 years age group (OR 2.74, p < 0.05). Despite low awareness levels, women paradoxically perceived themselves to be self-efficacious in seeking health care. Interestingly, just 49.1% Emirati women believed that good quality and affordable health care was available in the UAE. Only 28.8% of the participants believed there were sufficient female doctors to respond to health needs of women in UAE. Furthermore, only 36.7% Emirati women chose to be treated in the UAE over treatment in other countries.

**Conclusion:**

Emirati women clearly lack the knowledge on severity and vulnerability to heart disease in the region that is essential to improve cardiovascular related health outcomes. This study has identified the need for wider outreach that focuses on gender and age specific awareness on heart disease risks and symptoms. The study has also highlighted potential modifiable barriers in seeking health care that should be overcome to reduce morbidity and mortality due to heart disease among national women of UAE.

## Background

According to the World Health Organisation (WHO), cardiovascular diseases (CVD) cause more deaths globally than any other disease in the world [1]. CVD include a spectrum of disorders associated with the heart and blood vessels [1]. The American Heart Association (AHA) revealed that more than one in three adult women suffered from CVD and 8.6 million women around the world died due to CVD in 2002 [2, 3]. It has been recorded that 52% women die before reaching a hospital following a heart attack compared to 42% men [4].

Common symptoms of heart attack include pain in the centre of the chest and sweating, whereas non-specific symptoms include, pain in the arms, left shoulder, jaw and back, breathing difficulty, nausea, vomiting, lightheadedness, sleep disturbances and fainting [1, 5]. Women are more likely to suffer from the non-specific, atypical symptoms of heart disease than men [6]. Heart attacks usually happen in older women thus symptoms of heart disease may be masked by symptoms of chronic diseases, which could explain the delay in seeking health care and higher mortality following an ischaemic episode among women [5, 7].

Prevalence of hypertension and obesity in the Middle East is higher in women than in men; the region also claims the highest burden of diabetes [8]. Alternative forms of smoking, such as the water pipe smoking are becoming a popular social activity making it evident that women in the Middle East are at an increasing risk for heart disease [9]. The Weqaya cardiovascular screening programme under the patronage of Health Authority of Abu Dhabi similarly revealed high prevalence of cardiovascular risk factors in the population of UAE [10]. CVD accounted for 38% of all deaths in UAE in 2008 and was the leading cause of death among women in UAE during 2010 [11, 12]. This is contrary to the common belief that CVD primarily inflict men [12].

A study that analysed data on patients with acute coronary heart disease from 18 hospitals in UAE observed that women with heart disease were 13 years older than men [13]. Women presented with a higher number of risk factors compared to men. More women presented with atypical symptoms, however inability to recognise atypical symptoms, delayed diagnosis and late initiation of treatment may explain the higher rate of complications and in-hospital mortality among women. Mortality rate in UAE for women, following heart ailments was 4.6% compared to 1.2% for men [13].

Various studies have highlighted differences in presenting symptoms among males and females. Among women who had suffered a heart attack in the last 6 months, 95% described prodromal symptoms preceding the attack by up to a month [14]. Recognition of prodromal symptoms including unusual fatigue, sleep disturbances, breathlessness and anxiety could improve mortality rates among women [14]. The study, like many others highlighted that often women did not present with the common symptom of acute chest pain at all [6, 14–16]. More women described chest symptoms as fullness and pressure in the chest rather than pain in the centre or left of the chest, as men did [6, 16]. Goldberg et al. observed that the elderly and women were more likely to delay seeking health care than men and younger individuals, due to low perceived susceptibility to heart disease in women [15].

Winham and Jones [17] carried out a cross- sectional study on a convenience sample of 179 African American adults, using the AHA national survey questionnaire. Knowledge on heart disease was higher among females, older participants and those with higher education levels [17]. Chest pain was recognized as a symptom by 97%, however, atypical symptoms such as nausea was recognized by only 30% of the sample [17].

Trends in awareness were observed using AHA questionnaires in studies conducted in 1997 and 2005 using random samples in the United States [18, 19]. The latter study, in 2005 showed increased levels of awareness following national heart disease awareness campaigns [19]. Women who had received information on heart disease within the last year were more likely to modify their lifestyle to reduce cardiovascular risk factors. Magazines, television and physicians were considered the major sources of information although a larger number of older women listed newspapers and radio as a source of information compared to younger women [18, 19].

Heart disease awareness among women is an under researched subject worldwide, especially in the Middle East. There have been no studies related to heart disease awareness among women in the UAE. It is evident that there is a high prevalence of CVD risk factors in UAE. Women tend to have higher mortality and greater complications when suffering from heart disease, which could be ascribed to delayed diagnosis of heart disease. It is essential to have baseline knowledge of awareness levels and perceptions of the community involved to build a strong heart disease awareness and health promotion programme that focuses on women.

This study seeks to a) explore the awareness of heart disease among Emirati women and b) to understand Emirati women’s health care seeking behaviour in UAE.

## Methods

### Study design and sample recruitment

A cross sectional study was conducted to explore the awareness of heart disease among Emirati women and to recognise their health care seeking behaviour. A convenience sample of 706 participants filled out the questionnaire. Following data cleaning, incomplete questionnaires and those inadvertently filled out by men or non-UAE national women were excluded from analysis, which reduced the sample size to 676 Emirati women. Inclusion criteria required that the participants were female, UAE nationals (Emirati), above 18 years of age and have some formal school education. Men, non-Emiratis and women below 18 years of age were excluded.

### Data collection instrument and process

The questionnaire was adapted from the 2012 survey instrument used in the Women’s Health Study, carried out by the AHA. Permission to use the survey was obtained from the AHA [20]. Questions were added to increase relevance to the local context and to gain insight in to health care seeking behaviour among Emirati women.

The adapted questionnaire was a compilation of questions related to: awareness of heart disease, symptoms of heart disease and risk factors, action women would take on perceiving symptoms of heart disease, perceived susceptibility to heart disease and perceptions and preferences related to health care.

The questionnaire was translated to Arabic to ensure a wider participation from Emirati women. The questionnaire was piloted in a small group of eighteen Emirati women; minor modifications were made in the Arabic version to add clarity to the questions before the study was officially initiated. The survey was launched in Arabic and English through an online tool, ‘Survey Select’ along with an informed consent page, through various social media sites and some women organization websites. The informed consent sought consent for participation in the study and to allow publication of relevant findings from the study. Printed copies of the survey were made available to those who preferred them. The survey remained open between September 2014–December 2014.

Ethical clearance for the study was obtained from Zayed University’s Research Ethics Committee (UAE) (Ethics application number ZU14_041_F).

### Data management

Data were analysed using SPSS software. Age was dichotomized in groups roughly corresponding to a younger, pre-menopausal age group (18–45 years) and older, peri-menopausal (46–55 years) age group. Education levels were categorized in to three groups: high school, undergraduate level and graduate level or above. The seven emirates of UAE were placed in to four categories, Abu Dhabi, Dubai, Sharjah and ‘others’. Due to the low representation of participants from Ras Al Khaimah, Ajman, Ummul Quwwain and Fujairah data collected from residents of these emirates was combined under ‘other emirates’. Simple univariate frequencies were obtained. Pearson Chi square test of independence was used to observe the association of each variable with the emirate, age and education level of participants. A *p* value under 0.05 was considered significant*.*

Women, who scored 50% or above on the 38 questions related to knowledge of risk factors and symptoms of heart disease, were considered aware of heart diseases and its associated risk factors. Levels of awareness were compared to different emirates, age groups and education levels using the Pearson Chi square test of independence.

Binary logistic regression analysis was performed to find the odds of awareness of heart disease risk factors and symptoms in relation with emirates, age group and education levels. The analysis was carried out on a univariate model to give crude odds ratio (OR) with a confidence interval of 95% and a multivariate model to give adjusted odds ratio with a confidence interval of 95%.

### Methodological considerations

The findings of this study need to be considered in the light of various strengths and limitations. A cross sectional, descriptive study does not yield a causal inference, accordingly findings are limited to giving an approximation of the magnitude of the problem [21]. Use of a convenience sample, due to lack of access to a sampling frame for Emirati women, does not allow us to generalise the findings, nonetheless it does give an insight in to the levels of awareness and perspectives of Emirati women [22]. The adequacy of sample size and homogeneity of participants, all being Emirati women add strength to the findings [23]. There was a higher representation of women from the younger age group and from the two Emirates of Dubai and Abu Dhabi however these two emirates are also the larger and most populated Emirates of UAE [24].

The use of a pretested questionnaire fortifies the study; the AHA survey instrument has been used for many other studies [17–19] making it a reliable tool. All efforts were made to ensure the questionnaire was not too long and the questions were direct and culturally appropriate, this was verified through piloting questionnaire in both languages [22, 25]. The questionnaire was made available in multiple formats, via social media platforms, email and printed copies for wider participation. The availability of the survey instrument in both languages, Arabic and English allowed for representation of views in a language better understood by the participants.

## Results

### Demographics

A total of 676 self-identified Emirati women completed the questionnaire. Of these respondents 41.9% participants were from Abu Dhabi, 43.2% were from Dubai, 7.5% from Sharjah and 7.4% from the other Emirates of UAE. Majority of the participants, 81.7% had completed undergraduate education, 11.7% were high school educated and 6.7% had completed their graduate studies and beyond. A total of 97.3% participants were within the 18–45-year-old age group with only 2.7% being 46–55 years old. Only 3.6% participating women identified themselves with being afflicted by heart disease. Of the participants 70.1% were covered by health insurance. Health insurance had a significant relation with the emirate participants belonged to (*p* = 0.01)*,* with 89.8% women from Abu Dhabi having health insurance compared to 56.5% from Dubai, 54.9% from Sharjah and 54% from other emirates.

### Awareness of heart disease

**Fig. 1:**
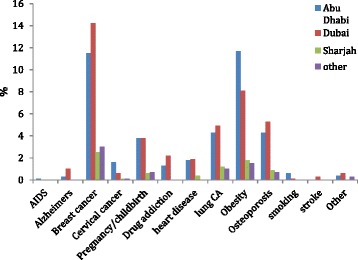
Greatest perceived health concern among Emirati women

Most participants, 31.2% highlighted breast cancer as the leading health concern for Emirati women, followed by obesity by 23.1%, lung cancer by 11.4%, osteoporosis by 11.2%, pregnancy by 9%, heart disease by 4% followed by drug addiction, cervical cancer, Alzheimer’s disease, smoking, stroke and AIDS **(**Fig. 1). No significant association was seen on comparison to emirates and age however the older age group considered osteoporosis as a bigger health concern than lung cancer.

In response to the question on leading cause of death in Emirati women, 32% of the women believed breast cancer was the leading cause of death while 31.4% participants considered heart disease to be the greatest cause of mortality, as shown in Table 1 **.** The result indicates that women did not perceive heart disease as their leading health concern yet paradoxically assumed it was one of the leading causes of mortality in Emirati women.

**Table 1:** Perceived leading cause of death in different Emirates

Leading cause of death	Abu Dhabi (*n* = 283)	Dubai (*n* = 292)	Sharjah (*n* = 51)	Other (*n* = 50)	Total (*n* = 676)
AIDs (%)	1 (0.1)	1 (0.1)	0	1(0.1)	3 (0.4)
Alzheimer’s Disease (%)	3 (0.4)	5 (0.7)	1 (0.1)	0	9 (1.3)
Breast Cancer (%)	80 (11.8)	103 (15.2)	17 (2.5)	16 (2.4)	216 (32.0)
Cervical Cancer (%)	13 (1.9)	11 (1.6)	1 (0.1)	6 (0.9)	31 (4.6)
Drug addiction (%)	0	1 (0.1)	0	0	1 (0.1)
Heart disease (%)	96 (14.2)	94 (13.9)	14 (2.1)	8 (1.2)	212 (31.4)
Lung Cancer (%)	4 (0.6)	4 (0.6)	1 (0.1)	1 (0.1)	10 (1.5)
Obesity (%)	30 (4.4)	23 (3.4)	6 (0.9)	2 (0.3)	61 (9.0)
Osteoporosis (%)	2 (0.3)	1 (0.1)	2 (0.3)	2 (0.3)	7 (1.0)
Pregnancy/childbirth (%)	22 (3.3)	20 (3.0)	7 (1.0)	9 (1.3)	58 (8.6)
Smoking (%)	2 (0.3)	1 (0.1)	0	1 (0.1)	4 (0.6)
Stroke (%)	19 (2.8)	23 (3.4)	2 (0.3)	2 (0.3)	46 (6.8)
Violence (%)	6 (0.9)	3 (0.4)	0	0	9 (1.3)
Other (%)	5 (0.7)	2 (0.3)	0	2 (0.3)	9 (1.3)
Total (%)	283 9(41.9)	292 (43.2)	51 (7.5)	50 (7.4)	676 (100)

**Fig. 2:**
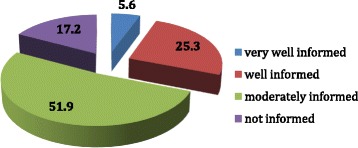
Perceived awareness levels (percentage) of heart disease among Emirati women

On stating self-perceived awareness on heart disease and risk factors, only 17.2% women felt they were not well informed about the disease. Of these participants, 24.1% were high school educated participants compared to 8.9% women with a graduate degree or higher. There was an indication that fewer women from the older age group perceived themselves to lack awareness on heart disease compared to the younger women Fig. 2.

The internet was considered a key source of information for health-related matters among 67.2% Emirati women. Friends and family were the main informants for 49.3% of the participants and television for 36.1% of the participants. Overall, only 17.8% participants indicated magazines as a source of information but there was a significant relationship showing that the older age group was more inclined to obtain information through magazines than younger age group (*p* = 0.01).

### Knowledge of symptoms of heart disease

**Fig. 3:**
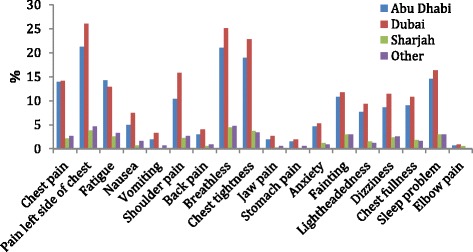
Recognition of symptoms of heart disease among Emirati women

From the 18 listed symptoms of heart disease on the questionnaire, participants could choose multiple symptoms that they associated with heart disease, Fig. 3 illustrates that overall 55.6% participants identified pain in the left side of the chest as a symptom, 55.3% selected shortness of breath as a symptom of heart disease. Tightness and pressure in chest was recognized by 48.8%, sleep disturbance identified by 36.8% and pain in centre of chest was acknowledged as a symptom in 32.7% of the participants. However, among the atypical symptoms, only 12% of the participants identified anxiety, 8.4% identified pain in the back, 6.1% highlighted vomiting, 5.5% recognised pain in jaw, 4.1% pain in stomach listed and 2.4% documented elbow pain (2.4%) as symptoms of heart disease.

Identification of shoulder pain and pain in jaw as symptoms of heart disease was significantly higher in the younger age group ((*p* = 0.001; *p* = 0.002), Awareness of shoulder pain was significantly higher in Dubai compared to all other emirates (*p* = 0.017).

Only 42% of the participants were aware that a heart attack could be possible without feeling chest pain, of these participants 40.5% belonged to the younger age group and only 1.5% belonged to the older age group.

### Knowledge of risk factors for heart disease

Recognition of some risk factors for heart disease was high: Obesity (75.1%), family history (63%), high blood pressure (62.7%) and high cholesterol (53.4%) being most widely recognised**.** Age (37%), diabetes (33.4%), smoking (37.4%) and menopause (6.5%) among others were surprisingly not widely stated as risk factors for heart disease.

Recognition of family history and high cholesterol as a risk factor was significantly higher in Dubai (*p* = 0.041). Identification of family history and stress as risk factors were significantly higher in the undergraduate group (*p* = 0.025; *p* = 0.011). Acknowledgment of family history (*p* = 0.001), male gender (p = 0.01), diabetes (*p* = 0.002) and high triglycerides (*p* = 0.037) as risk factors was significantly higher in the younger age group (*p* = 0.05) (Table 2).

**Table 2:** Association of knowledge of risk factors with age

Major Causes		18–45 Years		46–55 Years		*p*-value
		n	%	n	%	
Family history	No	245	36.2	0	0.0	0.001*
	Yes	413	61.1	18	2.7	
Obesity	No	162	24.0	6	.9	0.399
	Yes	496	73.4	12	1.8	
Age	No	414	61.2	12	1.8	0.745
	Yes	244	36.1	6	.9	
Male gender	No	576	85.2	10	1.5	<0.01*
	Yes	82	12.1	8	1.2	
Female gender	No	626	92.6	17	2.5	0.893
	Yes	32	4.7	1	0.1	
High blood pressure	No	244	36.1	8	1.2	0.524
	Yes	414	61.2	10	1.5	
Diabetes	No	444	65.7	6	.9	0.002*
	Yes	214	31.7	12	1.8	
Alcohol	No	522	77.2	13	1.9	0.464
	Yes	136	20.1	5	0.7	
Smoking	No	415	61.4	8	1.2	0.107
	Yes	243	35.9	10	1.5	
High cholesterol	No	310	45.9	5	0.7	0.105
	Yes	348	51.5	13	1.9	
High triglycerides	No	505	74.7	10	1.5	0.037*
	Yes	153	22.6	8	1.2	
Menopause	No	615	91.0	17	2.5	0.868
	Yes	43	6.4	1	0.1	
Not exercising	No	331	49.0	8	1.2	0.624
	Yes	327	48.4	10	1.5	
Stress	No	373	55.2	11	1.6	0.709
	Yes	285	42.2	7	1.0	
Stroke	No	517	76.5	16	2.4	0.290
	Yes	141	20.9	2	0.3	
Pregnancy	No	641	94.8	17	2.5	0.440
	Yes	17	2.5	1	0.1	
Others	No	618	91.4	17	2.5	0.927
	Yes	40	5.9	1	0.1	

### Perceived susceptibility and Self-efficacy to respond to heart disease

Only 10.5% respondents considered themselves at risk for heart disease. Identification of risk was significantly higher in women from Dubai compared to other emirates (*p* = 0.01)**.**

**Fig. 4:**
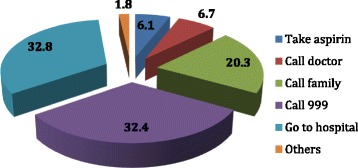
Action (in percentage) taken by Emirati women on suspecting a heart disease

In the event of a suspected heart attack Fig. 4 illustrates that majority of women believed they would go to a hospital themselves, or call the emergency number; many would call a family member for assistance. All women who assumed they would take aspirin for a heart attack belonged to the younger age group.

It was revealed that 69.5% of the participants knew how to call an ambulance, but a surprisingly high percentage of 30.5% women were incapable of calling for an ambulance. Of those who knew how to call for an ambulance, 67.3% belonged to the younger age group. Findings also suggest that Emirati women from Dubai and Abu Dhabi were more aware on how to call an ambulance compared to the other emirates.

When asked to consider whether women were as likely to seek medical treatment as men, 70.4% women agreed with the statement, indicating high perception of self-efficacy.

### Health care preferences and perceptions

Preference for consulting a regular doctor was shown by 50.4% participants. There was a suggestion that women from Abu Dhabi and from the older age group were more inclined towards consulting a regular doctor for their health concerns*.* Women from Dubai perceived they were significantly more able to visit the doctor when they felt the need to (*p* = 0.002) as shown in Table 3.

**Table 3:** Perceived self-efficacy to visit a doctor in association with Emirate

Response	Abu Dhabi	Dubai	Sharjah	other	Total
No (n, %)	133 (19.7)	119 (17.6)	25 (3.7)	35 (5.2)	312 (46.2)
Yes (n, %)	150 (22.2)	173 (25.6)	26 (3.8)	15 (2.2)	364 (53.8)
Total (n, %)	283 (41.9)	292 (43.2)	51 (7.5)	50 (7.4)	676 (100)

P = 0.002* using chi square test

Participants with undergraduate education showed significantly greater perceived ability to visit the doctor compared to other groups (*p* = 0.001).

A total of 49.1% Emirati women claimed quality health care was available in UAE at affordable prices. There was a significant relation between this perception and the emirate women belonged to (*p* = 0.01); people living in Abu Dhabi comprised most of those who felt affordable, quality health care was available in UAE.

The majority (66%) of Emirati women felt it was important to have female doctors tending to them, yet only 28.8% felt there were enough female doctors in UAE. The preference for female doctors was significantly lower in Dubai compared to women from other Emirates (*p* = 0.009).

Interestingly, 40.7% of the participants preferred to be treated in a foreign country instead of UAE. Only 36.7% participants would opt for treatment in UAE whereas 22.6% remained noncommittal. Responses varied significantly with age, with the older age group preferring treatment in the UAE (*p* = 0.0026)**.**

Most participants, 56.4% indicated a preference that the doctor should speak their first language, Arabic. Women from Sharjah and Abu Dhabi had a significantly higher predilection for doctors speaking their first language compared to Dubai (*p* = 0.005).

### Association of awareness with Emirates, education and age

Only 19.4% respondents (mean and standard deviation score of 21.81 ± 3.08), scored above 50% on the 38 questions related to awareness of heart disease, on the questionnaire and could be considered aware of heart disease and associated risk factors.

The emirate and age group women belonged to had a significant relation to their awareness levels (*p* = 0.004 and *p* = 0.034); women from Dubai and from the younger age group showed higher levels of awareness, depicted in Table 4. Education level did not have a significant relation with awareness levels**.**

**Table 4:** Awareness of heart disease in association with emirate, age and education levels

		Awareness about Heart Disease/risk factors				p-value
		No		Yes		
		n	%	n	%	
Emirate	Abu Dhabi	245	45	38	29	0.004*
	Dubai	218	40	74	56.5	
	Sharjah	42	7.7	9	6.9	
	Other	40	7.3	10	7.6	
Age	18–45 Years	534	98	124	94.7	0.034*
	46–55 Years	11	2	7	5.3	
Education	High school	63	11.6	16	12.2	0.417
	Undergraduate	449	82.5	103	78.6	
	Graduate or above	33	6.1	12	9.2	

*p < 0.05 considered significant using Pearson chi square test

### Binary Logistic regression analysis

Results from univariate models showed women in Dubai were 2.18 times as likely and 2.08 times as likely in the multivariate model to be aware of heart disease compared to women of Abu Dhabi (reference emirate). Odds of awareness in Sharjah and other Emirates were significantly higher than Abu Dhabi in both models as shown in Table 5.

**Table 5:** Odds estimation of levels of awareness in Emirati women using binary logistic regression

Parameter	n	Univariate	Multivariate
		OR (95% C.I)	OR (95% C.I)
Emirate			
Abu Dhabi	283	Reference	Reference
Dubai	292	2.18* (1.41,3.37)	2.08* (1.35, 3.22)
Sharjah	51	1.38 (0.62, 3.06)	1.34 (0.60, 3.03)
Other	50	1.61 (0.74,3.49)	1.61 (0.74, 3.50)
Age			
46–55 Years	18	Reference	Reference
18–45 Years	658	2.74* (1.04, 7.21)	2.11 (0.78, 5.72)
Education			
Graduate or above	45	Reference	Reference
Undergraduate	552	0.63 (0.31,1.26)	0.70 (0.34, 1.43)
High school	79	0.69 (0.29, 1.64)	0.73 (0.30, 1.75)

*Odds considered as significant with p < 0.05

It was also seen that the 18–45-year age group was 2.74 times more aware of heart disease and its risk factors than the 46–55-year age group, this was statistically significant on univariate analysis, however OR of 2.11 was not significant in the multivariate model. This result contradicts results obtained on perceived awareness, where older women had higher perceived awareness on heart disease. Levels of education did not show any significant association with awareness of heart disease and related risk factors.

## Discussion

The findings of the study are discussed considering the Health Belief Model (HBM), which explains the relation between health beliefs and health behaviours. People who deem they are at risk for a serious health problem are more likely to take action, if they believe action will reap benefits and they have the self-efficacy to take action [26].

It was evident from the findings that Emirati women in UAE did not consider heart disease to be a major health concern among women. Like Mosca et al.’s study, where women considered breast cancer to be a bigger health problem, this study also showed that only 4% Emirati women thought of heart disease as a major health concern [18]. African Americans believed obesity was the greatest health problem in the population, whereas Emirati women in this study considered obesity second to breast cancer as a health issue in women [17, 18]. Interestingly, despite not perceiving heart disease as a substantial health problem in Emirati women, it was considered the second highest cause for female mortality after breast cancer, as also seen in other studies [17–19].

Studies show awareness that heart disease is the leading health problem in women almost doubled following national awareness initiatives; highlighting the usefulness of nationwide awareness programmes [19]. It is even more intriguing to unearth that only 17.2% Emirati women believed they were not well informed about heart diseases, the remaining felt they were knowledgeable, thus perceived need for knowledge and consequently cues to action were considerably low among Emirati women.

Awareness of typical symptoms was much higher in Emirati women compared to the atypical symptoms, but on comparison with other studies where 97% and 67% participants identified chest pain as a symptom of heart disease, only 32.7% Emirati women identified pain in centre of chest while 55.6% identified pain on left side of chest as major symptoms [17, 18]. At least 10% of the women identified atypical symptoms in other studies compared to as few as 6.1% Emirati women identifying vomiting as a symptom of heart disease. Even fewer women were aware that pain in the jaw and elbow could be symptoms of heart disease [18]. This finding highlights the dearth of awareness of heart disease symptoms in Emirati women. Risk factor awareness was much higher than symptom awareness, 75.1% and 63.8% Emirati women identified obesity and family history respectively as risk factors, which is comparable to findings from Winham and Jones’ study [17].

Only 10.5% participants in our study perceived themselves as susceptible to heart disease, which is similar to observations in Lefler’s study [27]. A possible explanation for low perceived susceptibility is explained by the theory of social constructivism, whereby social and societal influences render women to be perceived as less susceptible than men to suffer from heart disease [28]. Comparatively low levels of awareness of atypical symptoms could be attributed to stereotypical media images of middle-aged men of falling to the ground with severe chest pain when suffering from a heart attack [27].

This study showed that the younger women were more knowledgeable on heart disease furthermore formal education levels did not influence awareness levels in Emirati women. These findings are in agreement with findings from the studies by Mosca et al., however negate findings in Winham and Jones’ study where higher levels of awareness were seen in the older and highly educated population [17–19]. Higher levels of awareness in younger age group could be justified by the increasing focus on preventive health care by the UAE government [10, 11]. However, another explanation could be the greater perceived need for awareness in this age group. These findings suggest effective measures are needed to target the older female population, who are more likely to suffer from heart disease yet are less aware of the need to seek more information [13]. Internet, social media, friends and family was the major source of information for most Emirati women. Older women relied on magazines for information, as also revealed in previous studies [18]. Given the high dependency on friends and family for information greater outreach for older women in UAE may be possible through community education and community participation in health promoting activities.

Another very significant finding revealed that most Emirati women would most likely go to the hospital themselves in an emergency or rely on family members to help. Only 32.8% participants felt they would call for emergency assistance on the first instance. This is a strong indication of the need for community members to recognise heart disease symptoms and know the optimal action to take in response to heart disease. Merely 6.2% women, of the younger age group claimed they would take aspirin if they felt an impending heart attack; this suggests rising awareness among younger women. Recent research has shown that aspirin is useful in men but considered ineffective in preventing a heart attack among women [5]. Thus, gender specific information related to heart disease is lacking, even in the more aware population.

Evidence of low self-efficacy and barriers to seeking treatment among Emirati women was apparent by the discovery that 30.5% Emirati women in the study did not know how to call for an ambulance. Self-efficacy and barriers to seeking treatment are often influenced by culture however perceived ability to seek health care assistance was considered the same in both genders by majority Emirati women. Despite awareness of obesity as a risk factor, exercise may not be culturally acceptable for Emirati women, where they are held to a preconceived image of feminism [29, 30]. Younger women, particularly those residing in the Emirate of Dubai considered themselves more self-efficacious in seeking health care. Older women preferred consulting a regular, female doctor yet there was an overall perceived dearth of female doctors. This could present as a barrier to seeking medical help among older Emirati women. Overall less than 50% participants believed good quality and affordable health care was available in UAE however residents of Abu Dhabi believed their health care to be better than women from other emirates. This could be rationalized by the ‘Thiqa Programme’ in Abu Dhabi, which provides free insurance coverage to all UAE nationals in the Emirate [31]. Despite cost not being a barrier to seeking treatment for Emiratis in UAE, majority would prefer to be treated in a country outside of UAE. These findings are in accordance with Winslow and Honein’s study, which revealed that women’s decisions related to health care were influenced by culture [29].

This study emphasizes low levels of awareness related to heart disease in all Emirati women in UAE, which explains reduced perceived severity and vulnerability. While many women believed, they were knowledgeable, findings from this study did not support their perceptions. Younger women, particularly in Dubai had a higher perceived self-efficacy compared to older women. Lack of female doctors, insufficient faith in health care in UAE, inability to recognise symptoms of heart disease, lack of awareness on how to call an ambulance and of the preferred emergency actions were identified as some barriers to seeking appropriate treatment. This study is important in recognising some modifiable factors, which could be directly related to the high rates of heart disease related mortality in Emirati women.

## Conclusion

Heart disease is a leading cause of mortality among women in the UAE. Women often present with atypical symptoms of heart disease, that are not well recognized, thus treatment are often delayed. UAE has a high prevalence of risk factors of heart disease, most of which are higher among women. Women need to be aware of the severity of heart disease in the region and their vulnerability to the disease to be able to act to improve cardiovascular related health outcomes. This knowledge is lacking among Emirati women of UAE. While levels of awareness levels seem higher in the Emirate of Dubai and among women belonging to the 18–45-year age group, there is a dire need for wider outreach to increase gender and age specific awareness of risk factors and symptoms of heart disease. Increased focus is required on raising awareness levels among women belonging to the older age group, who have a lower perceived need to acquire knowledge yet have lower levels of awareness and a greater risk of heart disease. This study has also identified potential barriers in seeking health care that should be overcome to translate knowledge in to action, which will ultimately reduce morbidity and mortality due to heart disease in Emirati women of UAE.

## Abbreviations

AHA: American Heart Association
CVD: Cardiovascular disease
HBM: Health Belief Model
OR: Odds ratio
UAE: United Arab Emirates
WHO: World Health Organisation
